# Supplementary material for: Fusion of Majorana bound states with mini-gate control in two-dimensional systems
Source: Nat Commun. 2022 Apr 1;13:1738. doi: 10.1038/s41467-022-29463-6 (PMC8976011; doi:10.1038/s41467-022-29463-6)
Supplement: Supplementary file 1 — Supplementary Information [file 41467_2022_29463_MOESM1_ESM.pdf]

Supplementary Information

Fusion of Majorana bound states with mini-gate control in two-dimensional systems

Tong Zhou,[1,*] Matthieu C. Dartiailh,[2] Kasra Sardashti,[2] Jong E. Han,[1]
Alex Matos-Abiague,[3] Javad Shabani,[2] and Igor Žutić[1,†]

[1]Department of Physics, University at Buffalo, State University of New York, Buffalo, NY 14260, USA
[2]Center for Quantum Phenomena, Department of Physics, New York University, NY 10003, USA
[3]Department of Physics and Astronomy, Wayne State University, Detroit, MI 48201, USA

Corresponding author emails: tzhou8@buffalo.edu; zigor@buffalo.edu

This file includes:

Supplementary Figures:

Supplementary Fig. 1: Calculated gate-controlled topological transition in an SJ.

Supplementary Fig. 2: Experimental gate-controlled topological transition in an SJ.

Supplementary Fig. 3: Spectrum evolution during the MBS fusion in an SJ.

Supplementary Fig. 4: MBS robustness against the deviation of the magnetic field from the junction interface.

Supplementary Fig. 5: SEM image for the fabricated VJ with 5 mini-gates.

Supplementary Fig. 6: Gate-controlled topological transition in a VJ.

Supplementary Fig. 7: Energy spectrum and charge densities for a VJ with a larger size.

Supplementary Notes:

Supplementary Note 1: Preparation of the initial states through initializations.

Supplementary Note 2: Protocol and spectrum evolution for the MBS fusion in a VJ.

Supplementary Note 3: Dynamical simulations for the MBS fusion in a VJ.

Supplementary Movies:

Supplementary Movie 1. An animation for the evolutions of the energy spectrum and wavefunction probabilities of the Majorana bound states during the nontrivial fusion in the straight Josephson junction with mini-gate control.

Supplementary Fig. 1: Calculated gate-controlled topological transition in an SJ

Supplementary Fig. 1 provides a gate-controlled evolution of the energy spectra, the probability density, ρ_P, and charge density, ρ_C, for the trivial and topological states in the SJ. Here, ρ_P and ρ_C are given by $\rho_P = |u|^2 + |v|^2$, and $\rho_C = e(|u|^2 - |v|^2)$, where e is the charge of the electron, while u and v are the particle and hole components of the wavefunction, respectively [1, 2]. For topological states, which are Majorana bound states (MBS), the wavefunctions are localized at the ends of the normal (N) region and the charge densities are vanishingly small and spread along the whole normal region. For the trivial states, the wavefunctions are more delocalized, while the charge densities are much larger. These results are in agreement with the previous work [3, 4], showing different properties of the trivial states and topological states.

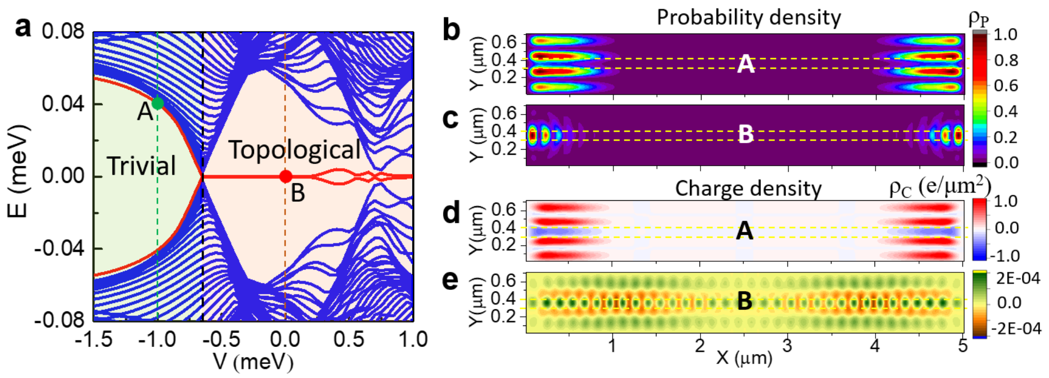

Supplementary Fig. 1. a The same as Fig. 4**a** in the main text, but with A (B) marking the lowest-energy states at V = -1 (0) meV in the trivial (topological) region. **b** and **c** Probability density, ρ_P, normalized to its maximum value, for the lowest-energy states (red lines) at V = -1.0 meV (marked as A) in the trivial region and V = 0 meV (marked as B) in the topological region, respectively. **d** and **e** The same as **b** and **c**, but for charge density, ρ_C. The parameters are taken from Fig. 4 in the main text.

Supplementary Fig. 2: Experimental gate-controlled topological transition in an SJ

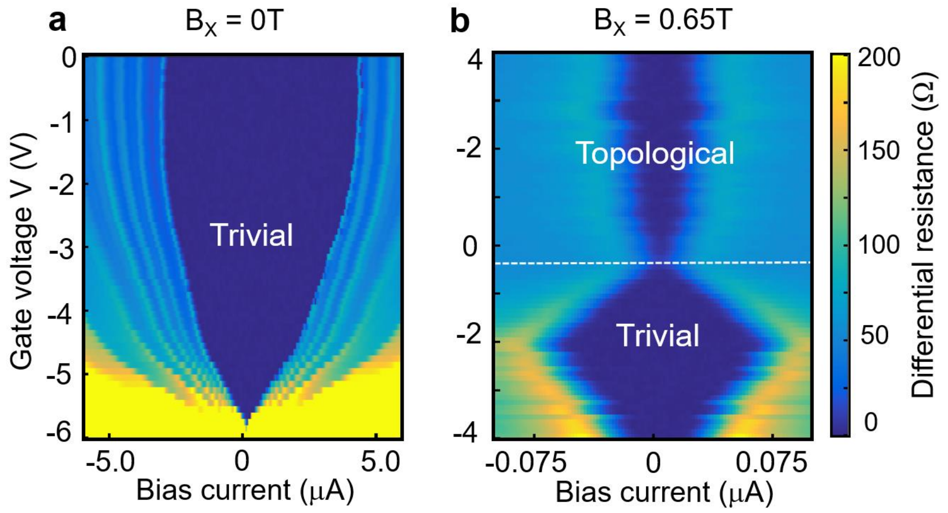

Supplementary Fig. 2. a Measured differential resistance for the SJ as a function of the applied gate voltage (V) with B_x = 0 T and **b** B_x = 0.65 T, consistent with the gap closing and reopening.

Supplementary Fig. 3: Spectrum evolution during the MBS fusion in an SJ

Supplementary Fig. 3 shows the spectrum evolution for the MBS fusion scheme in an SJ. The adiabatic evolution in the fusion scheme is verified by the calculated energy spectra evolution for the operations in nontrivial (A_1-A_4) and trivial (B_1-B_4) fusion. For any value of the continuously changing mini gates, the MBS are protected by the topological gap between the ground and first excited states which has the minimum value of ~6 μeV. The different signatures between the trivial and nontrivial fusions are verified by the calculated ρ_P and ρ_C of the fused MBS.

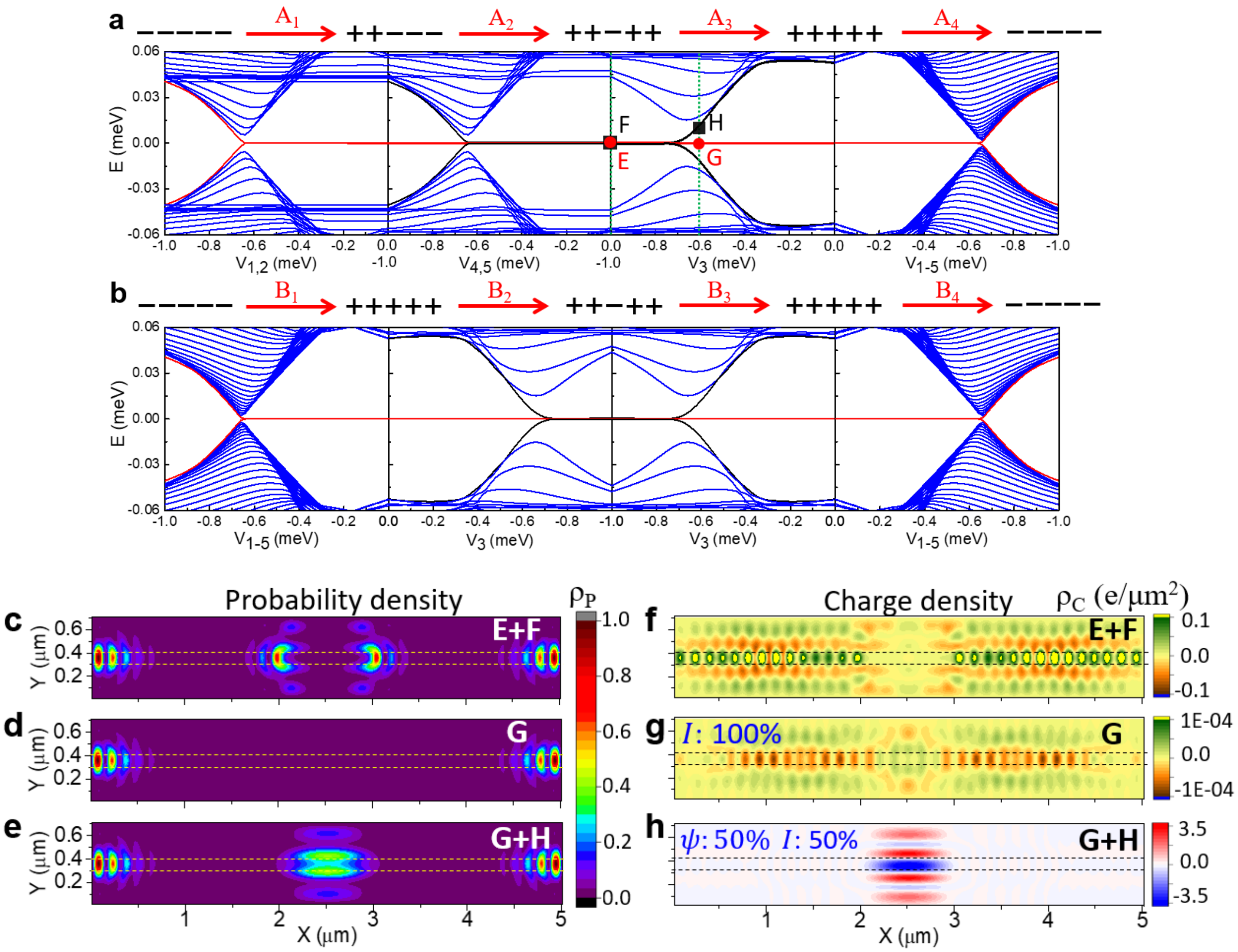

Supplementary Fig. 3. Calculated energy spectrum evolution for the operations in **a** nontrivial (A_1-A_4) and **b** trivial (B_1-B_4) fusion in an SJ, shown as a function of the relevant mini-gate voltage. Red and black lines: evolution of finite-energy states into MBS inside the topological gap. E and F indicate the two MBS pairs (degenerate ground states) in the + + − + + configuration (before fusion), while G and H indicate the ground and first excited state at V_3 = -0.6 meV in the + + + + + configuration (after fusion). **c** Sum of the probability densities, ρ_P, for E and F. **d-e** ρ_P for G and sum of the ρ_P for G and H. **f-h** The same as **c-e**, but shown for charge densities, ρ_C. The dashed line mark the N regions covered by the mini gates. The (minimum, maximum) values in **f**, **g** and **h** are (-0.07, 0.09), (-0.00009, 0.00004) and (-3.5, 2.9), respectively. The parameters are taken from Fig. 4 in the main text.

Supplementary Fig. 4: MBS robustness against the deviation of the magnetic field from the junction interface.

Supplementary Fig. 4 shows the robustness of the MBS against the misalignment angle, θ, between the N/S interface and the applied in-plane magnetic field. Our calculations reveal that topological superconductivity is supported for $\theta \leq 0.15\pi$.

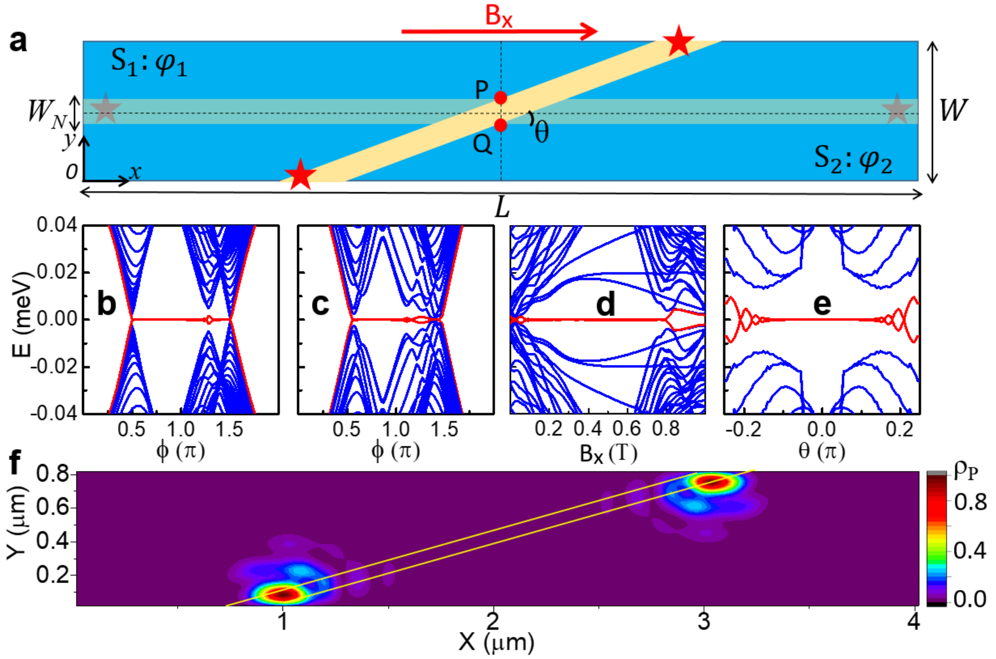

Supplementary Fig. 4. a Schematic of a tilted junction with a misalignment angle θ from the applied B_x. MBS (stars) reside at the opposite ends of the N region (yellow). **b** Energy spectra for an SJ with B_x = 0.4 T as a function of the phase difference, $\phi = \varphi_1 - \varphi_2$. **c** Same as **b** but for a tilted junction with θ = 0.1π. **d** Energy spectra for a tilted junction, ϕ = π and θ = 0.1π as a function of B_x. **e** Energy spectra for a tilted junction, ϕ = π and B_x = 0.4 T as a function of θ. **f** Probability density, ρ_P, for the lowest (red) energy states with ϕ = π, θ = 0.1π and B_x = 0.4 T. When rotating the N region, we fix the P and Q points with the coordinates of $x_{P,Q}$ = L/2 and $y_{P,Q} = (W \pm W_N)/2$. The geometric parameters are L = 4 μm, W = 0.8 μm and W_N = 0.1 μm. The other parameters are taken from Fig. 4 in the main text.

Supplementary Fig. 5: SEM image for the fabricated VJ with 5 mini-gates.

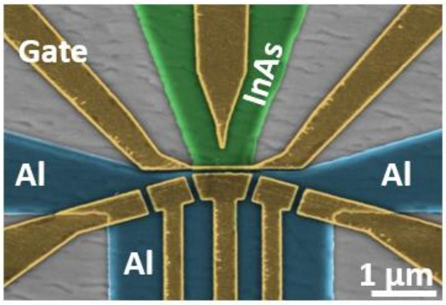

Supplementary Fig. 5. SEM image of a fabricated VJ with 5 mini-gates.

Supplementary Fig. 6: Gate-controlled topological transition in a VJ

Supplementary Fig. 6 provides gate-controlled evolution of the energy spectra, ρ_P, and ρ_C for the trivial and topological states in the VJ. For topological states, the wave functions are localized at the ends of the N region, while the charge densities are vanishingly small and spread along the whole N region. For the trivial states, the wavefunctions are more delocalized, while the charge densities are much larger.

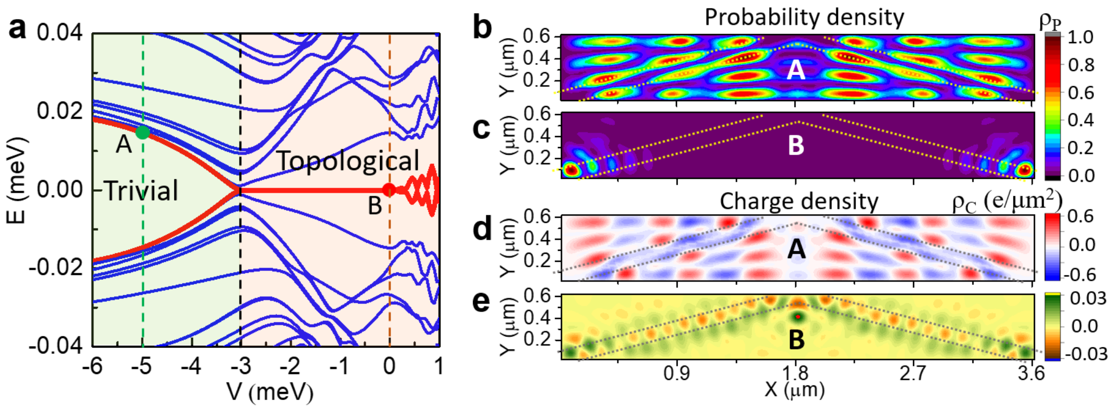

Supplementary Fig. 6. a The same as Fig. 6**c** in the main text, but with A (B) marking the lowest energy states at V = -5 (0) meV in the trivial (topological) region. **b** and **c** Probability density, ρ_P, normalized to its maximum value, for the lowest energy states (red lines) at V = -5.0 meV (marked as A) in the trivial region and V = 0 meV (marked as B) in the topological region, respectively. **d** and **e** The same as **b** and **c**, but for charge densities, ρ_C, respectively. The parameters are taken from Fig. 6 in the main text.

Supplementary Fig. 7: Energy spectrum and charge densities for a VJ with a larger size.

Supplementary Fig. 7 shows that the oscillations of the zero-energy modes can be reduced when the system size is increased. Correspondingly, ρ_C of the zero-energy modes decreases drastically.

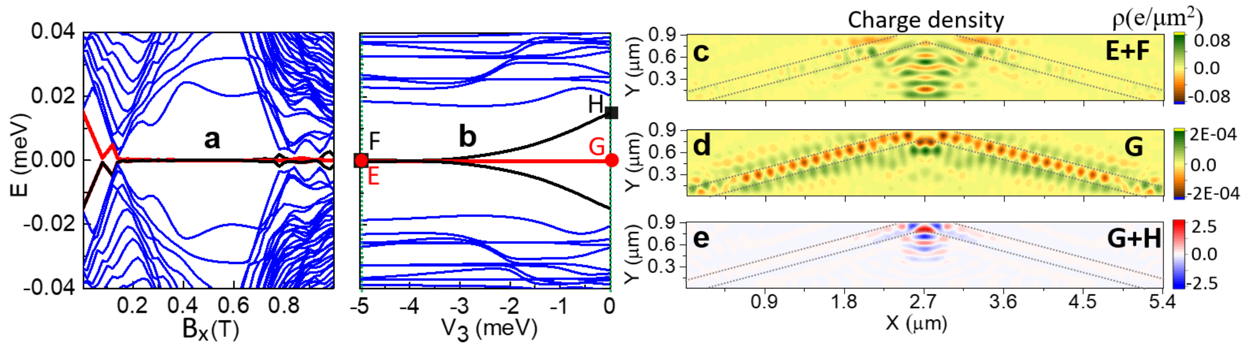

Supplementary Fig. 7. a The same as Fig. 6**f** in the main text, but with L = 5.4 μm and W = 0.9 μm. **b** The same as the energy spectrum of A_3 in Fig. 7**a** in the main text, but with L = 5.4 μm and W = 0.9 μm. **c**-**e** The same as Figs. 7**e**-**g** in the main text, but with L = 5.4 μm and W = 0.9 μm. The (minimum, maximum) values in **c**-**e** are (-0.06, 0.08), (-0.00019, 0.00019) and (-2.2, 2.5), respectively. The dashed lines indicate the normal regions covered by the mini gates. The parameters are taken from Fig. 6 in the main text.

Supplementary Note 1: Preparation of the initial states through initializations

For a two-fermions system (f_{12} and f_{34}) discussed in the manuscript, there are four typical occupation states, depending on whether they are occupied ($|1\rangle$) or unoccupied ($|0\rangle$), i.e. $|00\rangle$, $|11\rangle$, $|10\rangle$, and $|01\rangle$. Based on the fusion rules given in Fig. 1 in the main text, irrespective of the initial state, the trivial fusion cannot change the fermion occupations, while the nontrivial fusion can always induce a superposition of the occupied and unoccupied states, having 50% probability to change the fermion occupations. As a result, we should observe charge fluctuations when repeating the nontrivial fusion, while no charge fluctuations when repeating the trivial fusion.

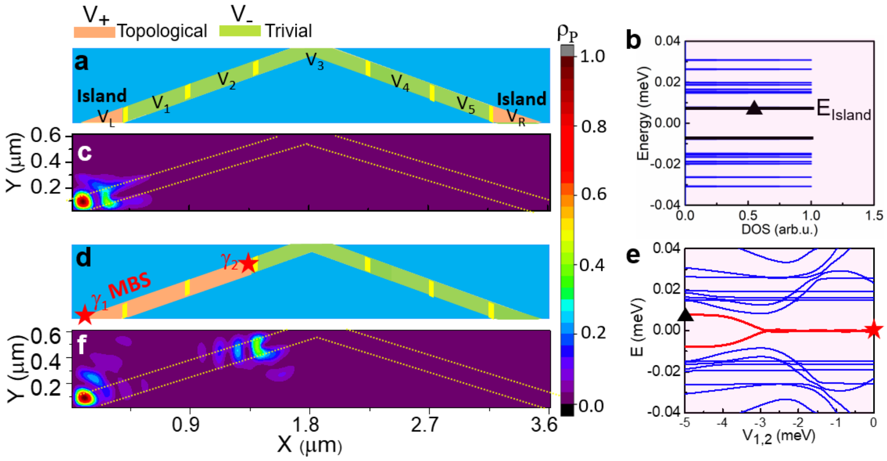

Supplementary Fig. 8. a Schematic of the VJ having five mini gates with voltages V_1-V_5 and two additional half-length mini gates, with voltages V_L and V_R. We set $V_{L,R} = V_+ = 0$ (orange) and $V_{1\text{-}5} = V_- = -5$ meV (green) to engineer the two short mini-gates into the effective quantum islands. **b** Density of states for **a** with $V_L = V_+$ but $V_R = V_-$. **c** Probability density, ρ_P, of the E_{Island} marked by the triangle in **b**, supporting the formation of the quantum island. Schematic **d** and the spectrum evolution **e** when the topological region is extended by changing V_1 and V_2 from V_- to V_+. **f** ρ_P of the lowest positive energy marked by the star in **e**, supporting the MBS formation. The parameters are taken from Fig. 6 in the main text.

However, if each time the initial state changes randomly, the trivial fusion may also give charge fluctuations (due to trivial change of the initial occupations), bringing possible false signature of the fusion rules. To overcome this problem, it is important to keep the same initial occupation in every fusion cycle. To prepare the initial occupations of f_{12} and f_{34}, we first need to generate two quantum islands to store the f_{12} and f_{34}. Such islands should form at the edge of the system to be accessible and flexibly controlled. Considering that our MBS are initially generated at the two VJ ends, we propose to create two quantum islands by adding half-length mini gates (L, and R), with voltages V_L and V_R at the ends of VJ, as shown in Supplementary Fig. 8a. Considering the length of the L (R) is small, when it is in the topological region, it can behave as an effective quantum island with the lowest energy level, E_{Island}, within the superconducting gap. Taking the quantum island L as an example, we calculate its energy spectrum and find the $E_{Island} \sim 8\mu V$ as shown in Supplementary Fig. 8b. Our calculated probability density (Supplementary Fig. 8c) confirms the effective quantum island indeed forms at L. Such an island can store the fermion f_{12}. When the E_{Island} is occupied (unoccupied), the f_{12} is in $|1\rangle$ ($|0\rangle$) state. By changing the V_1 and V_2 from V_- to V_+ (Supplementary Fig. 8d), the topological region can be extended, and the f_{12} in quantum island adiabatically evolves into MBS ($f_{12} = \gamma_1 + i\gamma_2$), supported by the calculated spectrum evolution (Supplementary Fig. 8e). Since it is an adiabatic evolution, the f_{12} does not change its occupation until the nontrivial MBS fusion happens. Similarly, the fermion f_{34} can be stored at the quantum island R, and its occupation ($|1\rangle$ or $|0\rangle$) can be well defined

depending on whether its E_{Island} is occupied or not. Combing the occupations of f_{12} and f_{34}, we have four possible typical initial states ($|00\rangle$, $|11\rangle$, $|10\rangle$, and $|01\rangle$).

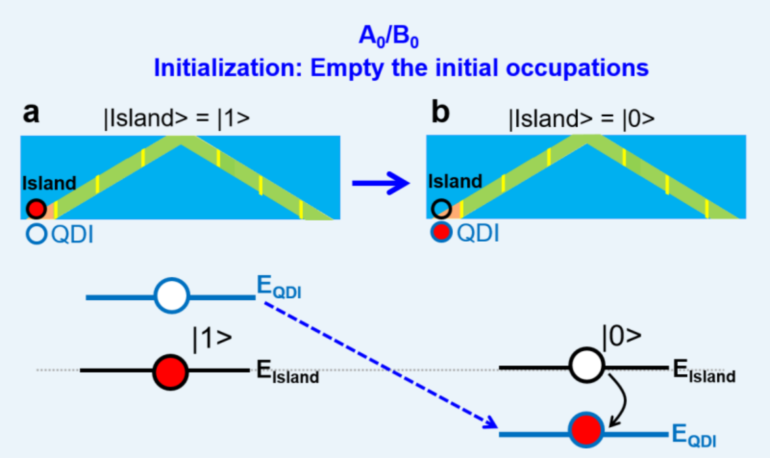

Supplementary Fig. 9. Schematic for the A_0/B_0 operation of "initialization". **a** The initial occupation of the fermion $f_{12} = \gamma_1 + i\gamma_2$ in the quantum island. If the f_{12} is unoccupied ($|0\rangle$), it is our desired state and the initialization does not do anything. Thus, here we only show the case when f_{12} is occupied ($|1\rangle$), where the lowest positive energy level of the L quantum island ($E_{Island} \sim 8\mu eV$ in Supplementary Fig. 8b) must be occupied. We couple the initializing QD, QDI (blue circle) with energy $E_{QDI} > E_{Island}$, to the quantum island. **b** By shifting $E_{QDI} < E_{Island}$, the charge of f_{12} in the quantum island can transfer into the QDI, leaving a $|0\rangle$ state for f_{12}. Similarly, the $|0\rangle$ state for f_{34} can be prepared by coupling another QD to the quantum island.

In principle, the initial state should be $|00\rangle$ because it is the ground state of the system. However, if for any reason the initial state is different (for example $|11\rangle$, $|10\rangle$, or $|01\rangle$ state), we can still empty it through additional QD by performing initialization procedure. Such initialization (A_0/B_0) shown in Supplementary Fig. 9 is added before the MBS generation and manipulation (A_{1-4}/B_{1-4}) in our fusion protocol (Supplementary Fig. 10), which can empty the initial occupations to give an initial state $|00\rangle$. We couple an additional initializing QD, QDI (Supplementary Fig. 9a), as a reservoir to receive the occupied fermion in the L quantum island and drive f_{12} into the $|0\rangle$ state. An advantage of using the QDI is that we can flexibly control its energy and coupling with the quantum island by gating [5]. Now, let us see how the QDI helps to empty the quantum island. For the $|0\rangle$ state, it is our desired state and the initialization operation does nothing. For the $|1\rangle$ state, by initially making the highest unoccupied energy level of the QDI, $E_{QDI} > E_{Island}$ (Supplementary Fig. 9a), and then making the $E_{QDI} < E_{Island}$ (Supplementary Fig. 9b), the charge of f_{12} in the L island can transfer into the QDI, leaving a $|0\rangle$ state for f_{12}. Similarly, we can drive f_{34} into the $|0\rangle$ state. After such an initialization, f_{12} and f_{34} are in the desired $|00\rangle$ state. Then we decouple the QDI with the VJ to make sure the QDI does not perturb the MBS manipulations in the VJ.

Supplementary Note 2: Protocol and spectrum evolution for the MBS fusion in a VJ

With the initialization, the initial VJ state is prepared as $|00\rangle$. Then we can perform A_1-A_4 (B_1-B_4) operations to implement the MBS nontrivial (trivial) fusion as shown in Supplementary Fig. 10. The spectrum evolution of A_1-A_4 and B_1-B_4 is shown in Supplementary Fig. 11. We can see two quantum island states can adiabatically evolve into two MBS pairs, where their pair configurations depend on the A_{1-2}/B_{1-2} operations. The operation A_3 fuses the MBS from different pairs and gives a nontrivial fusion, accessing both vacuum, I, and an unpaired fermion, ψ, with 50% probability, while the B_3 trivially fuses the MBS from the same pairs, corresponding to I with 100% probability. After A_3/B_3, we use the QPC to detect the QD charge

number, Q_{QD}. For the trivial fusion, the Q_{QD} is 0; while for the nontrivial fusion, the Q_{QD} is 0 or 1e with the same probability, giving an expectation value of 0.5e. Such a different fusion outcome is supported by our dynamical simulations in Supplementary Fig. 12. After the QPC charge sensing detection, we perform A_4/B_4 operations to reset the system. By repeating such A_0-A_4 (B_0-B_4) operations, we can repeat the MBS fusion with the same $|00\rangle$ occupation, where every time the fusion occurs (the system goes to + + + + + after A_3/B_3), we use the QPC to detect the Q_{QD}. The detected current, I_{QPC}, through the QPC is denoted by I_{TF} (I_{NF}) for the trivial (nontrivial) fusion. While the expected I_{TF} remains the same, the I_{NF} fluctuates during the fusion cycles. To suppress the possible trivial background charge fluctuation, we can focus on the difference, ΔI_F, between I_{TF} and I_{NF}. Measuring such a fluctuating ΔI_F is a direct evidence for the non-Abelian statistics of MBS.

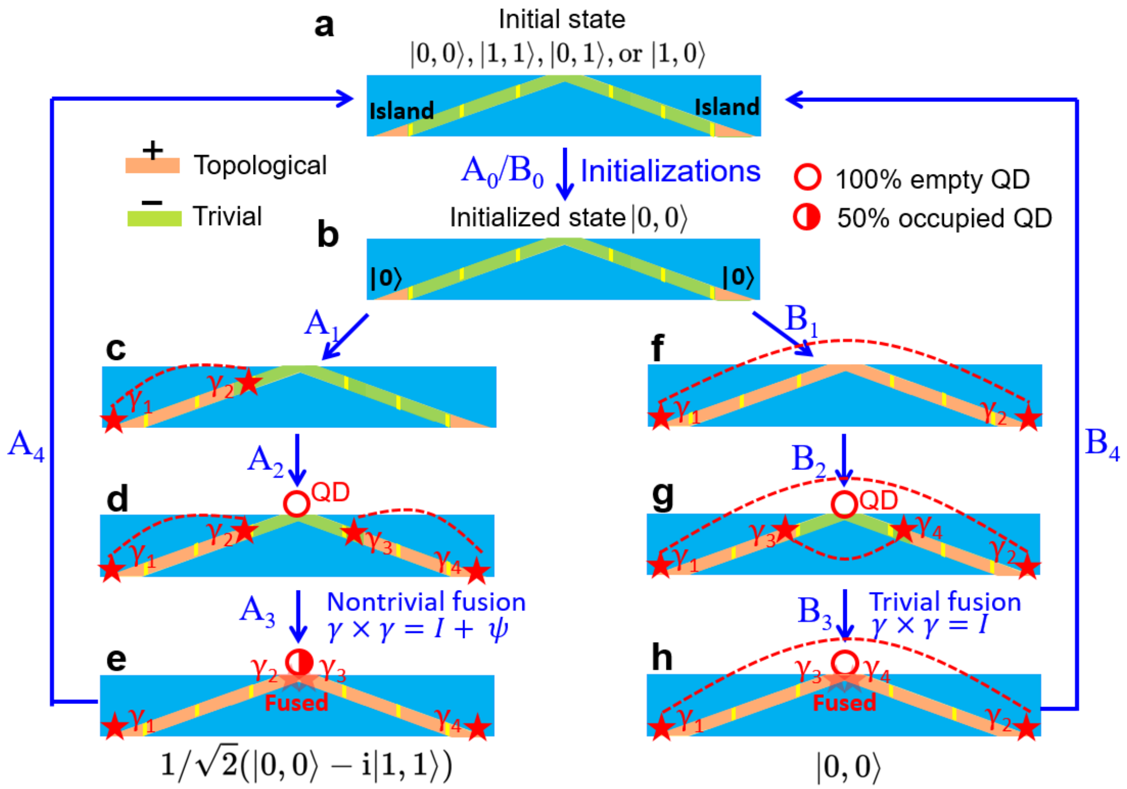

Supplementary Fig. 10. Probing non-Abelian statistics through MBS fusion in a VJ coupled with a detection QD. The scheme is supported by the calculated probability and charge densities in Fig.7 in the main text, spectrum evolution in Supplementary Fig. 11, as well as the dynamical simulations in Supplementary Fig. 12. The stars indicate MBS, and the dashed lines link the same MBS pair. **a** Initial trivial state with four typical occupations ($|00\rangle$, $|11\rangle$, $|01\rangle$, or $|10\rangle$) in − − − − − mini gates with two quantum islands. **b** A_0/B_0: Initialization as shown in Supplementary Fig. 9, where the initial state $|00\rangle$ is prepared. **c** A_1: changing − − − − − to + + − − −, MBS pair (γ_1, γ_2) is created. **d** A_2: changing + + − − − to + + − + +, a second MBS pair (γ_3, γ_4) is created. Then coupling a QD at the apex and making the QD empty. **e** A_3: changing + + – + + to + + + + +, the MBS (γ_2, γ_3) are nontrivially fused at the apex. As a result, the occupation changes into the superposition of $|00\rangle$ and $|11\rangle$, accessing both vacuum, I, and an unpaired fermion, ψ, with 50% probability. After A_3, the QPC shown in Fig. 6 in the main text is used to detect the QD charge number, with the expected same probability for charge zero and one. (**f**) B_1: changing − − − − − to + + + + +, the MBS pair (γ_1, γ_2) is created. **g** B_2: changing + + + + + to + + – + +, a second MBS pair (γ_3, γ_4) is created. Then coupling the QD at the apex and making the QD empty. **h** B_3: changing + + – + + to + + + + +, the MBS (γ_3, γ_4) are trivially fused and the occupation is the same as the initial $|00\rangle$, corresponding to I with 100% probability. After B_3, the QPC is used to detect the QD charge number and zero charge is expected. A_4/B_4: Resetting the system: changing + + + + + to − − − − −, and

decoupling the VJ with the QD. As a result, the system returns to the initial two quantum island states. MBS fusion can be repeated following such operations.

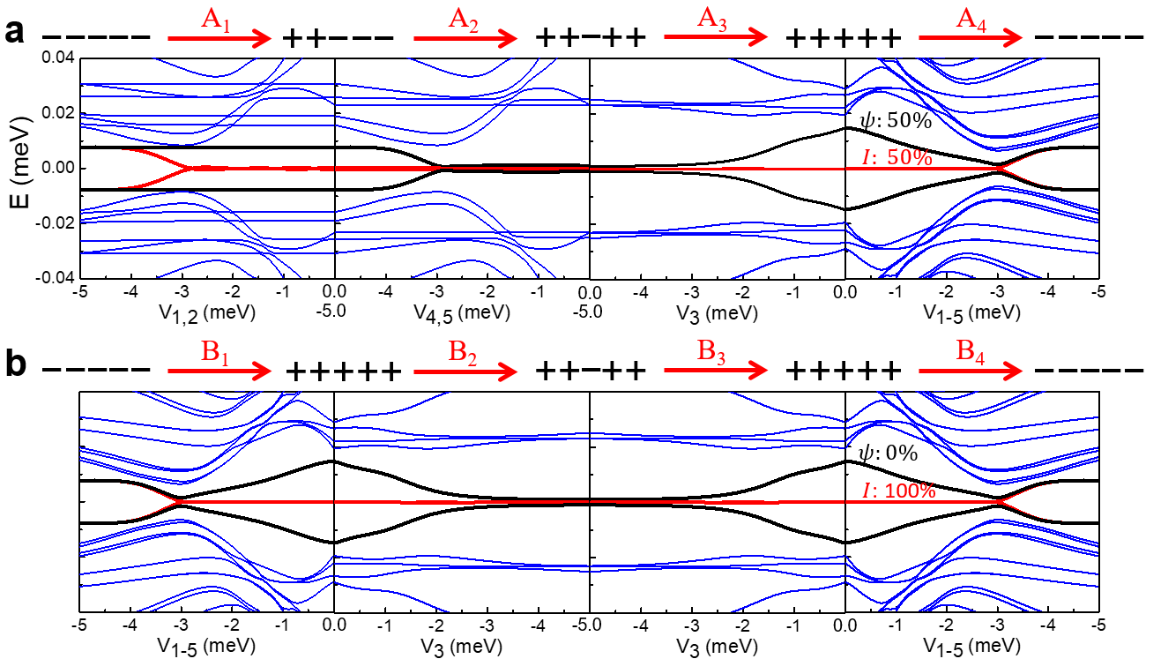

Supplementary Fig. 11. Calculated energy spectrum evolution for the operations of **a** nontrivial (A_1-A_4) and **b** trivial (B_1-B_4) fusion for a VJ in Supplementary Fig. 10, shown as a function of the relevant mini-gate voltage. Red and black lines: evolution of finite-energy states into MBS inside the topological gap.

Supplementary Note 3: Dynamical simulations for the MBS fusion in a VJ

With the QD coupled to the VJ, the Hamiltonian for the system is $H = H_{VJ} + H_{QD} + H_{V\text{-}Q}$. Here, the H_{VJ} [given by Eq. (2) in the main text] describes the VJ. For simplicity, we suppress the spin index in the following terms. $H_{QD} = E_{QD} d^\dagger d$ describes the QD with a gate-tunable energy level E_{QD}, where d^+ (d) creates (annihilates) an electron in the QD. $H_{V-Q} = \sum_{i\in\text{apex}} (\lambda c_i^\dagger d + H.C)$ describes the coupling between the QD and VJ apex, where $c_i{}^\dagger$creats an electron in the VJ and λ is the coupling strength. To elucidate the fusion outcomes and reveal how they can be detected by the QD charge sensing, we perform dynamical simulations based on the time-dependent Bogoliubov–de Gennes equation [6, 7]

$$i\hbar \frac{\partial}{\partial t} \psi(t) = H(t)\psi(t),$$

where $\psi(t)$ is the quasiparticle wave function in the Nambu representation. The evaluation of the wave function during a time Δt is given by $\psi(t + \Delta t) = U(t + \Delta t, t)\psi(t)$ with the time-evolution operator

$$U(t + \Delta t, t) = \tau \exp\left[-i \int_t^{t+\Delta t} d\xi \, H(\xi)\right],$$

where τ is the switching time to control the evolution speed. For a sufficiently short Δt, the time-evolution operator is approximated as $U(t + \Delta t, t) \approx \exp[-iH(t)\Delta t]$.

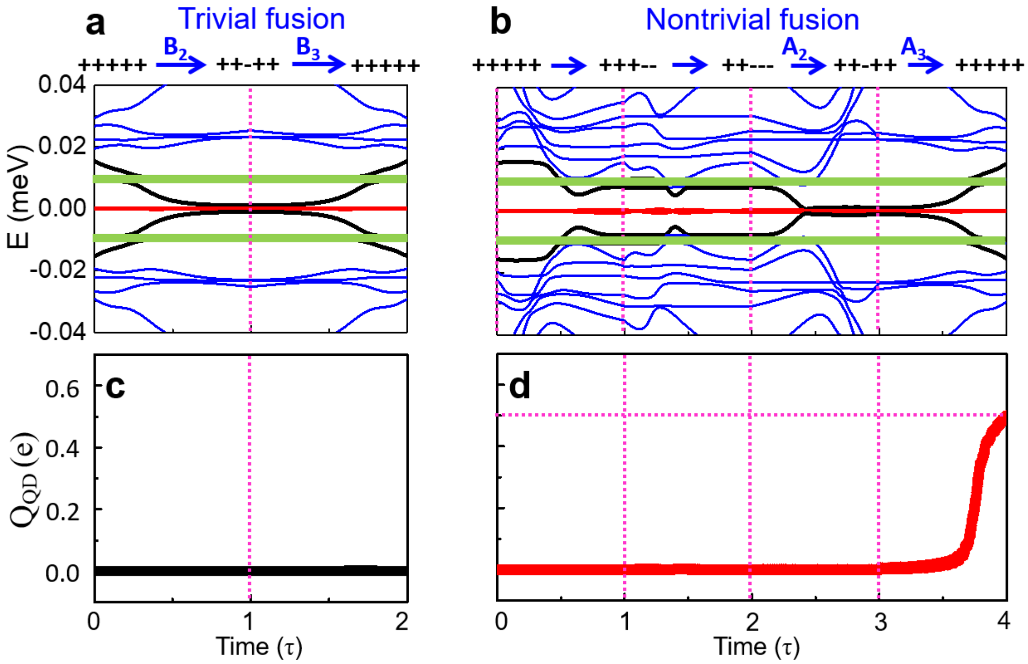

Supplementary Fig. 12. a-b Calculated spectrum evolution and **c-d** corresponding charge average of the QD, Q_{QD}, during the nontrivial and trivial fusion processes with a cyclic loop. The green lines in **a** and **b** indicate the energy levels of the QD. The parameters of E_{QD} = 10 μeV, λ = 3 μeV, and τ = 2.4 ns are used for dynamical simulations. Other parameters are taken from Fig. 7 in the main text.

We show how the QD affects the spectrum evolution during the fusion in Supplementary Figs. S12 a and b. To estimate the QD charge change after trivial and non-trivial fusions, we perform a time evolution for the initial state$|00\rangle$, corresponding to all negative (positive) states being occupied (empty). Details about the preparation of the $|00\rangle$ state are discussed in Supplementary Note 1. For adiabatic processes and low energy excitations, only the four states with energies $\{-E_2, -E_1, E_1, E_2\}$ (ordered from smaller to larger) closer to zero are involved in the fusion process. During the trivial fusion, the occupancy of these states remain unchanged. However, during the non-trivial fusion the occupancy of the two negative energy states is transferred, with 50% probability to the positive energy states. In our scheme, the QD level is set to the final value of E_2, allowing to detect the final charge transferred into this, initially empty, energy state. Since both E_1 and E_2 are initially empty and the occupancy transfer from level $-E_2$ to E_2 is forbidden by particle-hole symmetry, the final charge measured in the QD can only originate from the initially occupied state ψ_{-E_1}. The average charge in the QD can be estimated as $Q_{QD} = e \times P_{-E_1\to E_2} \times P_{E_2\to QD}$, where $P_{-E_1\to E_2}$ is the probability for the occupancy of level $-E_1$ to be transferred to E_2 and $P_{E_2\to QD}$ is the probability that the charge in level E_2 tunnels into the QD. If the process is adiabatic, the outcome of trivial and non-trivial fussions are, $P_{-E_1\to E_2} \to 0$ and $P_{-E_1\to E_2} \to \frac{1}{2}$, respectively. The time-dependence of the QD charge can therefore be estimated as $Q_{QD}(t) = e \times \left|\langle\psi_M(t)|\psi_{QD}\rangle\right|^2$, where $\psi_M(t)$ describes the time evolution of the energy level $-E_1$ [i.e., $\psi(0) = \psi_{-E_1}$] and ψ_{QD} is the wavefunction of the QD.

For the trivial fusion, the MBS ψ_M (γ_1, γ_2) is first created in the + + + + + configuration. Taking it as an initial state, we perform dynamical simulations following the sequence of B_2 [creating (γ_3, γ_4)] and B_3 [fusing (γ_3, γ_4)] as shown in the fusion protocol (Supplementary Fig. 10). The calculated Q_{QD} remains zero (Supplementary Fig. 12c), indicating the 100% fusion channel *I* in the trivial fusion. For the nontrivial fusion, (γ_1, γ_2) is created in the + + – – – configuration. To clearly see the difference between the trivial and nontrivial fusion, it is important to perform the dynamical simulations from the same initial state in both

cases. To achieve this, starting from the same initial ψ_M as that in the trivial fusion, we first adiabatically move (γ_1, γ_2) from + + + + + + to + + – – – – configuration and then follow the sequence of A_2 [creating (γ_3, γ_4)] and A_3 [fusing (γ_2, γ_3)] to finish the nontrivial fusion process as shown in Supplementary Fig. 12b. In contrast to the trivial fusion, the calculated Q_{QD} is rising up after the nontrivial fusion occurs (Supplementary Fig. 12d), indicating there is a finite probability that the QD receives an unpaired fermion from the VJ. Furthermore, the maximum Q_{QD} = 0.5e is consistent with the expected equal probabilities of channels *I* and ψ in the nontrivial fusion. Such different fusion outcomes from the dynamical simulations clearly demonstrate the non-Abelian statistics of the MBS fusion.

[1] Alicea, J. New directions in the pursuit of Majorana fermions in solid state systems, *Rep. Prog. Phys.* **75**, 076501 (2012).

[2] Scharf, B. & Žutić, I. Probing Majorana-like states in quantum dots and quantum rings, *Phys. Rev. B* **91**, 144505 (2015).

[3] Pientka, F. et al. Topological superconductivity in a planar Josephson junction. *Phys. Rev. X* **7**, 021032 (2017).

[4] Hell, M., Leijnse, M. & Flensberg, K. Two-dimensional platform for networks of Majorana bound states. *Phys. Rev. Lett.* **118**, 107701 (2017).

[5] Hanson, R., Kouwenhoven, L. P., Petta, J. R., Tarucha, S. & Vandersypen, L. M. Spins in few-electron quantum dots. *Rev. Mod. Phys.* **79**, 1217-1265 (2007).

[6] Amorim, C. S., Ebihara, K., Yamakage, A., Tanaka, Y. & Sato, M. Majorana braiding dynamics in nanowires. *Phys. Rev. B* **91**, 174305 (2015).

[7] Sanno, T., Miyazaki, S., Mizushima, T. & Fujimoto, S. Ab initio simulation of non-Abelian braiding statistics in topological superconductors. *Phys. Rev. B* **103**, 054504 (2021).
