# Supplementary material for: Fusion of Majorana bound states with mini-gate control in two-dimensional systems
Source: Nat Commun. 2022 Apr 1;13:1738. doi: 10.1038/s41467-022-29463-6 (PMC8976011; doi:10.1038/s41467-022-29463-6)
Supplement: Supplementary file 2 — Description of additional Supplementary File [file 41467_2022_29463_MOESM2_ESM.pdf]

Description of Additional Supplementary Information files

Supplementary Movie 1. An animation for the evolutions of the energy spectrum and wavefunction probabilities of the Majorana bound states during the nontrivial fusion in the straight Josephson junction with mini-gate control.
