# Supplementary figures and images for: Fusion of Majorana bound states with mini-gate control in two-dimensional systems
Source: Nat Commun. 2022 Apr 1;13:1738. doi: 10.1038/s41467-022-29463-6 (PMC8976011; doi:10.1038/s41467-022-29463-6)

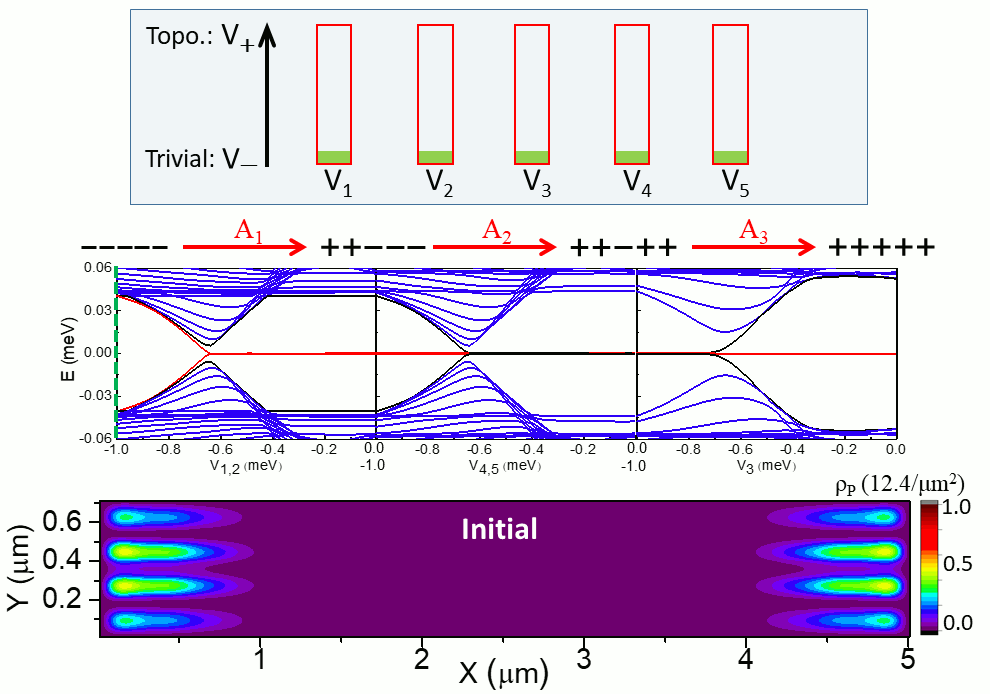

Supplement: Supplementary file 3 — Supplementary Movie 1 [file 41467_2022_29463_MOESM3_ESM.gif]
